# *Propionibacterium acnes* Is an Unusual Cause of Acute Scrotal Abscess in a Preterm Infant

**DOI:** 10.1155/2017/7942707

**Published:** 2017-05-23

**Authors:** Naoto Nishizaki, Tadaharu Okazaki, Yu Adachi, Kaoru Obinata, Hiromichi Shoji, Toshiaki Shimizu

**Affiliations:** ^1^Department of Pediatrics, Juntendo University Urayasu Hospital, Chiba, Japan; ^2^Department of Pediatric Surgery, Juntendo University Urayasu Hospital, Chiba, Japan; ^3^Department of Pediatrics, Juntendo University Faculty of Medicine, Tokyo, Japan

## Abstract

Acute scrotal abscess is an extremely rare condition in neonates and may mimic testicular torsion. Most of these abscesses have reportedly been due to* Staphylococcus* and* Salmonella *spp. infections. We herein report a unilateral acute scrotal abscess in a preterm infant born at 26 weeks in whom* Propionibacterium acnes* was isolated from the collected scrotal fluid culture. To our knowledge, this is the first case report implicating* P. acnes* as a causative agent of neonatal scrotal abscess. Based on such findings,* P. acnes* infection should be considered in differential diagnosis of acute scrotal abscess in neonates, particularly in preterm infants.

## 1. Introduction

Although scrotal abscess is rare and uncommon in neonates, especially in preterm infants, differential diagnosis of acute scrotal swelling should be considered [1]. Neonatal cases of scrotal abscess were reported to be unilateral, with most of them caused by* Staphylococcus* and* Salmonella *spp. [2]. However, there have also been other case reports of scrotal abscess implicating microorganisms, such as beta hemolytic* Streptococcus*,* Bacteroides*,* Escherichia coli*, and* Klebsiella *spp. [3–6]. Bacteria usually enter the skin through a crack or an injury [1, 2]. Only a single reported case of neonatal scrotal abscess occurring secondary to aerobic and anaerobic bacterial infection has been described before; the cause of this abscess was undetermined [4]. We herein describe a 1-month-old preterm-born boy with an unusual case of unilateral scrotal abscesses caused by* Propionibacterium acnes* which is a Gram-positive pleomorphic and anaerobic bacterium. To our knowledge, this is the first case of neonatal acute scrotal abscess caused by* P. acnes* that was associated with immunosuppression.

## 2. Case Presentation

A male infant at 26 2/7 weeks of gestational age (birth weight, 759 g) was delivered by cesarean delivery to a 32-year-old healthy woman due to nonreassuring fetal status. After delivery, the baby's Apgar scores were 2 and 5 at 1 and 5 min., respectively. Tracheal intubation was performed soon after the delivery, and mechanical ventilation was initiated immediately after admission to the Division of Neonatal Intensive Care Unit (NICU). In addition, 120 mg of a surfactant agent was administered for respiratory distress syndrome. Although mechanical ventilation continued, his general condition was stable until three weeks after birth. At 23 days of age, he suddenly presented with pitting edema, hypotension (30/15 mmHg), and oliguria, and laboratory results indicated hyponatremia (Na: 125 mEq/L) and hyperkalemia (K: 6.0 mEq/L). Hypotension was treated with hydrocortisone (5 mg/kg/dose). After hydrocortisone administration, his symptoms dramatically improved. Patient met the clinical criteria for late-onset circulatory collapse (LCC), which responds to glucocorticoid therapy but not to volume expansion or vasopressors, and hydrocortisone administration was scheduled for two weeks (2–5 mg/kg/day). The patient's vital signs were stable, and abdominal and scrotal examination was unremarkable until 36 days of age.

**Figure 1:**
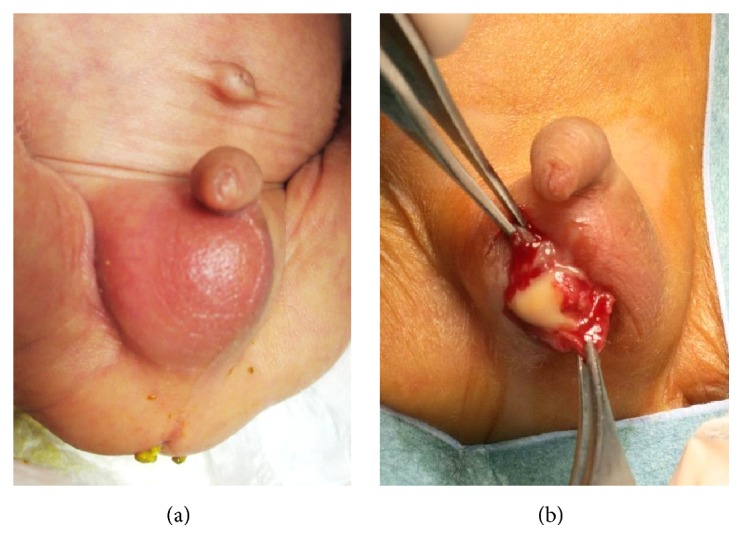
(a) Right scrotum was swollen and indurate. Upon physical exam, a palpable 1-cm hard structure was present in the right scrotum. (b) Surgical exploration revealing purulent fluid on the right testis.

At the age of 37 days, the right side of the scrotum was swollen and indurate; with no history of trauma, this was suggestive of an infection. Upon physical examination, a palpable 1-cm hard structure was present in the right scrotum, as shown in Figure 1(a). Inguinoscrotal ultrasonography was performed with a high-frequency linear probe and color Doppler to assess vascularity. Renal and urinary tract ultrasonography was normal. On the same day, the recorded laboratory data were a white blood cell count (WBC) of 25,200/mm^3^ (normal value < 12,000/mm^3^) and a C-reactive protein (CRP) level of 1.1 mg/dL (normal value < 0.3 mg/dL). From these data, acute scrotal abscesses and/or unilateral testicular torsion were suspected. After consultation with pediatric surgeons, urgent surgical exploration through separate hemiscrotal transverse incision was performed.

Observed during surgery, the right tunicae vaginalis was markedly thickened and contained pus (Figure 1(b)). The testis and epididymis appeared enlarged and hyperemic and were covered by thick necrotic, fibrinous exudate. Careful inspection showed no torsion of the spermatic cords. Attempts to cannulate the peritoneal cavity through the processus vaginalis failed, suggesting that this was not patent. The purulent tunical fluid was drained, and a sample was cultured. Local debridement and irrigation were performed, and meropenem (40 mg/kg per day) was added to his antibiotic therapy for 7 days. Resolution of the scrotal swelling occurred in 5 days. Although his initial blood and urine cultures were sterile,* P. acnes* was isolated from the scrotal fluid culture obtained during surgery. Antimicrobial susceptibility testing revealed that this strain was susceptible to meropenem [minimum inhibitory concentration (MIC) < 2 *μ*g/mL] and ampicillin (MIC < 2 *μ*g/mL). Therefore, for deescalation of the antimicrobial agent, his meropenem therapy was changed to ampicillin. Further antibiotic therapy normalized CRP and WBC levels. The postoperative course was uneventful. At the time of drafting this case report, he is currently being followed up by the Divisions of NICU and Pediatric Surgery.

## 3. Discussion

To our knowledge, this is the first report of a neonatal scrotal abscess caused by* P. acnes* during hospitalization and under steroid administration for LCC. As aforementioned, acute scrotal abscess is an extremely rare condition in neonates. Raveenthiran and Cenita reported nine cases of scrotal abscess, in which* Staphylococcus *spp. was most commonly isolated [2]. Huang and Chuang described a scrotal infection caused by* Salmonella enteritidis*, and in their review, they reported five other cases in infants younger than 3 months due to the same pathogen [7]. As stated above, beta hemolytic* Streptococcus*,* Bacteroides*,* E. coli*, and* Klebsiella *spp. were also reported to cause scrotal abscess in neonates [3–6]. However, our case is the first of a premature infant who developed scrotal abscess due to* P. acnes*.* P. acnes* is part of the skin and mucous membrane commensal microbial flora [8] and is mainly located in the pilosebaceous follicles and external auditory canal. It also presents a particular tropism for the conjunctiva, oral cavity, and intestines. This bacterium is frequently an opportunistic pathogen, sustaining infectious inflammatory processes in various organs and tissues [9, 10].

A review of the available literature indicated that intrastromal colonization of* P. acnes* is rare. The only evidence present in the literature is a report by Yamamoto et al. that described the onset of an intrascrotal and vesicular granuloma likely sustained by* P. acnes* in an elderly patient [9]. In our case, we speculated that the immunosuppressive state due to hydrocortisone administration for LCC may have subsequently led to a* P. acnes* infection. Generally, this bacterium is sensitive to a large variety of antibiotics (*β*-lactam antibiotics, carbapenem, macrolides, glycopeptides, and tetracyclines), highly resistant to metronidazole, and weakly sensitive to treatment with aminoglycosides [10].

Conversely, managing acute scrotal swelling in neonates can be challenging, with scrotal infections often resembling testicular torsions. Neonatal scrotal abscesses often have none of the recognized signs of inflammation, and this limits the value of clinical assessment at this age [2]. Moreover, because the initial symptoms are quite similar, it is often difficult to differentiate infectious pathologies of the scrotum from testicular torsion by clinical examination, imaging, and, at times, even surgical exploration. Although ultrasound examination of the scrotum in these circumstances is often performed, the sensitivity and specificity are too low to achieve the correct diagnosis [1]. Therefore, surgical exploration may be necessary to avoid missing a possibly correctable condition, such as torsion of the testis or strangulated hernia [3]. The differential diagnosis herein was made by using laboratory and screening tools in combination with surgical exploration. Surgical drainage of the abscess and systemic antibiotic therapy were curative in our case. However, since both blood and urine cultures were negative, the pathway of invasion of this anaerobic organism is still unknown. In the future, when recurring genitourinary tract infection occurs, cystourethrography should be performed to investigate congenital anomalies.

In summary, our case highlights the first premature infant to develop scrotal abscesses caused by* P. acnes* and has gleaned insight into this rare neonatal condition. In case of swelling of newborn scrotum, it is imperative to distinguish between emergency acute scrotal abscess and testicular torsion. Therefore, neonatologists should consider such symptoms in male infants presenting with intractable scrotal abscess, albeit rare. In addition, in the case of newborn infants who are immunosuppressed by steroid administration,* P. acnes* should be considered as a causative agent in scrotal abscess, particularly in preterm infants.
